# Molecular survey of *Enterocytozoon bieneusi* in sheep and goats in China

**DOI:** 10.1186/s13071-016-1304-0

**Published:** 2016-01-19

**Authors:** Ke Shi, Mengjie Li, Xiaoxing Wang, Junqiang Li, Md Robiul Karim, Rongjun Wang, Longxian Zhang, Fuchun Jian, Changshen Ning

**Affiliations:** College of Animal Science and Veterinary Medicine, Henan Agricultural University, Zhengzhou, 450002 China

**Keywords:** *Enterocytozoon bieneusi*, Goats, Sheep, ITS gene

## Abstract

**Background:**

Over recent years, several studies have conducted genotyping of *Enterocytozoon bieneusi* in various hosts worldwide using sequence analysis of the ribosomal internal transcribed spacer (ITS), however, relatively little is known about *E. bieneusi* in sheep and goats in China. Therefore this research was conducted to understand the prevalence and genotype distribution of *E. bieneusi* in farmed sheep and goats in China.

**Results:**

A total of 1025 fecal specimens from farmed animals in various geographic areas were collected. Overall, PCR and sequence analysis of the ITS detected *E. bieneusi* in 34.4 % (353/1025) of isolates; of which the prevalence in goats was 28.8 % (176/611) and in sheep was 42.8 % (177/414). Phylogenetic analysis of ITS sequences identified 42 genotypes (nine known and 33 novel ones). These consisted of four known genotypes (D, KIN-1, EbpC, and F) and 10 novel genotypes (CHG6, CHG7, CHG9, CHG19, CHG23, CHG25, CHS5 and CHS10–CHS12) which all belonged to the so-called zoonotic group 1. A further four known genotypes (CD6, COS-I, BEB6, and J) and 22 novel genotypes (CHG1–CHG3, CHG5, CHG8, CHG10–CHG14, CHG16–CHG18, CHG20, CHG22, CHG24, CHS3, CHS4 and CHS6–CHS9) formed a clade within the group 2. One novel genotype (CHG21) was clustered in the group 9 with the genotype CM4.

**Conclusions:**

*E. bieneusi* is highly prevalent, widely distributed, and genetically diverse in Chinese farmed goats and sheep. Some of the genotypes identified are potentially zoonotic.

**Electronic supplementary material:**

The online version of this article (doi:10.1186/s13071-016-1304-0) contains supplementary material, which is available to authorized users.

## Background

Microsporidia are a diverse group of obligate intracellular pathogens related to fungi [1]. To date, approximately 1300 species belonging to 160 microsporidian genera are known to infect invertebrate and vertebrate hosts, including humans [2, 3]. Among the 14 microsporidian species infectious to humans, *Enterocytozoon bieneusi* is regarded as the most commonly identified microsporidian species in humans [4, 5]. Thus far, in addition to humans, *E. bieneusi* has been detected in domestic animals such as cattle, sheep, goats, rabbits, pigs, dogs, cats, and horses [6–14], and in wild animals such as raccoons, muskrats, beavers, foxes, otters, nonhuman primates (NHPs), llamas, guinea pigs, deer and snakes [15–21] and birds [22].

*E. bieneusi* spores are morphologically indistinguishable and *in vitro* cultivation methods for this pathogen have not been developed. Therefore, molecular methods are required to identify genotypes within the species that infect humans, animals or both [3]. Until now, the internal transcribed spacer (ITS) of the rRNA gene has been used widely for species identification and genotyping of *E. bieneusi* isolates [11, 23, 24] and more than 200 *E. bieneusi* ITS genotypes have been identified [3–12]. Previous studies have reported that some genotypes are host-specific, whereas others are zoonotic because of their capability to infect both humans and animals [15, 16, 25–28]. Currently, over 30 countries have been surveyed to gather data on the molecular epidemiology of *E. bieneusi* in humans and other animals [29]. In China, this pathogen has been detected in HIV-positive and -negative patients, children, non-human primates (NHPs), dogs, cats, pigs, cows, sheep, goats, deer and snakes [8, 9, 11, 12, 20, 21, 30–37]. Moreover, this parasite has been identified in wastewater in several Chinese cities [38]. However, few studies have been conducted on *E. bieneusi* infections in sheep and goats in China [37]. In the present study, a large number of farms were surveyed to gain a better understanding of the prevalence and genotype distribution of *E. bieneusi* in sheep and goats in various parts of China.

## Methods

### Ethics statement

This research was conducted according to the Chinese Laboratory Animal Administration Act (1988) after review and approval of its protocol by the Research Ethics Committee of Henan Agricultural University. Appropriate permission was obtained from the farm owners before collection of fecal specimens from their sheep and goats.

### Fecal specimen collection

A total of 1025 fresh fecal samples were collected from sheep and goats in Henan, Heilongjiang, Liaoning, Yunnan, Anhui, and Shaanxi Provinces and Chongqing city between March 2011 and October 2013 (Table 1 and Additional file 1). Specimens were collected either directly from the rectum or were collected in sterile plastic containers during defecation (without the fecal sample having contact with the ground). One fecal specimen per sheep or goat was collected in a container marked with the breed, gender, age, and feeding habits of the animal. No obvious clinical symptoms were observed during specimen collection, which were then transported in an icebox to the laboratory for examination.

**Table 1:** Prevalence and genotype distribution of *E. bieneusi* isolates from goat and sheep farms from different Provinces

Species	Geographic source	No. of farms	No. of specimens	No. (%) of positive specimens	ITS genotype(s) (*n* ^a^)
Goats	Henan Province	10	343	113(32.9)	BEB6(22), E(3), F(2), KIN-1(2), D(2), J(1), COS-I(1), CD6(4), CHG1(13), CHG2(6), CHG3(10), CHG5(3), CHG6(1),CHG7(1),CHG8(1), CHG9(1), CHG10(1), CHG11(1), CHG13(1), CHG18(1), CHG20(1), CHG21(1), CHG22(1), CHG23(1) ), CHG25(1);
	Yunnan Province	4	134	30(22.4)	BEB6(14), E(4), F(1), D(1), COS-I(1), CD6(1), CHG1(1), CHG3(2), CHG5(1), CHG16(1), CHG17(1), CHG19(1);
	Anhui Province	1	80	6(7.5)	BEB6(1), CHG5(1), CHG3(1);
	Chongqing city	1	8	5(62.5)	CHG1(2), CHG3(1), CD6(1), CHG12(1);
	Shaanxi Province	1	46	22(47.8)	BEB6(4), E(1), F(1), CHG1(3), CHG3(3), CD6(3), CHG5(2), CHG14(1), CHG16(1), CHG24(1);
	Total	17	611	176(28.8)	BEB6(41), D(3), E(8), F(4), KIN-1(2), J(1), CHG1(19), CHG2(6), CHG3(17), CD6(9), CHG5(7), CHG6(1), CHG7(1), CHG8(1), CHG9(1), CHG10(1), CHG11(1), CHG12(1), CHG13(1), CHG14(1), COS-I(2), CHG16(2), CHG17(1), CHG18(1), CHG19(1), CHG20(1), CHG21(1), CHG22(1), CHG23(1), CHG24(1), CHG25(1) ;
Sheep	Henan Province	4	310	161(51.9)	BEB6(53), COS-I(12), CM4(1), CHG3(5), CHS3(2), CHS4(1), CHS5(1), CHS6(1), CHS10(1), CHS12(1);
	Liaoning Province	1	64	6(9.4)	BEB6(3);
	Heilongjiang Province	1	40	10(25.0)	BEB6(4), COS-I(2), CHS7(1), CHS8(1), CHS9(1), CHS11(1);
	Total	6	414	177(42.8)	BEB6(60), COS-I(14), CHG3(5), CM4(1), CHS3(2), CHS4(1), CHS5(1), CHS6(1), CHS7(1), CHS8(1),CHS9(1) CHS10(1), CHS11(1), CHS12(1);
All the Total		23	1025	353(34.4)	F(4), KIN-1(2), J(1), CHG1(19), CHG2(6), CHG3(22), CD6(9), CHG5(7), CHG6(1), CHG7(1), CHG8(1),CHG9(1) CHG10(1), CHG11(1), CHG12(1), CHG13(1), CHG14(1), COS-I(16), CHG16(2), CHG17(1),CHG18(1), CHG19(1), CHG20(1), CHG21(1), CHG22(1), CHG23(1), CHG24(1), CHG25(1), CM4(1), CHS3(2), CHS4(1), CHS5(1), CHS6(1), CHS7(1), CHS8(1), CHS9(1), CHS10(1), CHS11(1), CHS12(1), BEB6(101), D(3), E(8)

*n* ^a^, number of genotypes

### Specimen preparation

The fecal specimen from each container was transferred to a sterile beaker immediately on arrival at the laboratory, soaked for thirty minutes at room temperature in 30 ml of tap water, and then stirred with a clean stick to ensure thorough mixing. Each mixture was then put through a 7.62-cm-diameter sieve (pore size 45 μm) and concentrated by centrifugation at 3500 r · min^-1^ for 5 min. The concentrated fecal material was stored in 2.5 % potassium dichromate solution at 4 °C prior to DNA extraction.

### DNA extraction and PCR amplification

The stored fecal specimens were washed three times with distilled water and genomic DNA was extracted by using an E.Z.N.A.® Stool DNA kit (Omega Biotek Inc., Norcross, GA, USA) according to the procedure recommended by the manufacturer. The extracted DNA was stored at −20 °C before it was used for PCR amplification. DNA from each specimen was tested at least three times for *E. bieneusi* DNA using a nested PCR assay that amplified a 392 bp product that encompassed the terminal part of 18S rRNA gene, the complete internal transcribed spacer (ITS), and a partial region of 5.8S rRNA gene [15]. The KOD-Plus-Neo amplification enzyme (Toyobo Co. Ltd., Osaka, Japan) was used for PCR amplification. Nonacetylated bovine serum albumin (Solarbio Co. Ltd., Beijing, China) (final concentration 400 ng/L) was used to neutralize PCR inhibitors during the primary PCR. The secondary PCR products were examined by agarose gel electrophoresis and visualized after GelRed (Biotium Inc., Hayward, CA) staining.

### Sequencing and phylogenetic analysis

Since some fecal samples were collected from the same animals, positive specimens from different farms, animals, species and genders were selected for sequencing. The selected secondary PCR products were purified using Montage PCR filters (Millipore, Bedford, MA) and sequenced using a BigDye Terminator v3.1 cycle sequencing kit (Applied Biosystems) on an ABI 3730 DNA analyzer (Applied Biosystems, Foster City, CA). The nucleotide sequences were confirmed by bidirectional sequencing and by sequencing a new PCR product if necessary. The sequences obtained were aligned with reference sequences downloaded from GenBank using the ClustalX 2.0 (http://www.clustal.org/) program to determine the genotypes. Genotypes from this study were compared with known *E. bieneusi* ITS genotypes by neighbor-joining analysis of the aligned *E. bieneusi* sequences using Mega 5.0 (http://www.megasoftware.net/). Bootstrap analysis was used to assess the robustness of clusters using 1000 replicates. A previously established nomenclature system was used for naming the *E. bieneusi* ITS genotypes [24].

### Statistical analysis

Infection rates were compared using the chi-square test and a difference was considered statistically significant when the *P* value was <0.05. The analysis was done using QuickCalcs software (GraphPad Software Inc., La Jolla, CA).

### Nucleotide sequence accession numbers

Representative nucleotide sequences from this study have been deposited in GenBank under accession numbers KP262357–KP262398.

## Results

### Prevalence of *E. bieneusi* in goats and sheep

Of the 1025 fecal specimens from goats and sheep, overall 353 specimens (34.4 %) were *E. bieneusi*-positive by PCR amplification of the ITS locus. The prevalence in goats was 28.8 % (176/611) and in sheep was 42.8 % (177/414). The differences in the infection rates between sheep and goats were statistically significant different (χ^2^ = 21.27, *p* < 0.01). For goats, *E. bieneusi* was found on 17 of the studied farms from four provinces and one city (Table 1 and Additional file 1). The prevalence ranged from 7.5 % to 62.5 % for each goat farm, with significant differences in prevalence between different goat farms(χ^2^ **=** 73.21, *p* < 0.01). The prevalence rates were 62.5 % in goats from Chongqing City, 47.8 % in Shaanxi Province, 32.9 % in Henan Province, 22.4 % in Yunnan Province, and 7.5 % in goats from Anhui Province (χ^2^ **=** 35.81, *p* < 0.01). For sheep, *E. bieneusi* was detected in six farms from three provinces. The prevalence varied from 9.4 % to 67.4 % in the studied sheep farms, and the differences in infection rates were statistically significant (χ^2^ **=** 67.84, *p* < 0.01) (Table 1 and Additional file 1). The prevalence were 51.9 %, 25.0 %, and 9.4 % in sheep from Henan Province, Heilongjiang Province, and Liaoning Province, respectively (χ^2^ **=** 44.96, *p* < 0.01) (Table 1).

There were no statistically significant differences in the prevalence among the age groups in goats (χ^2^ = 4.50, *p* > 0.05) and sheep (χ^2^ = 5.61, *p* > 0.05) (Table 3). Likewise, there were no statistically significant differences for gender in goats (χ^2^ = 0.01, *p* > 0.05) and sheep (χ^2^ **=** 2.05, *p* > 0.05) (Table 2). There were some differences in prevalence between grazing and household goats, with a higher prevalence in household goats (χ^2^ **=** 5.75, 0.01 < *p* < 0.05) (Table 2), but there was no significant difference in prevalence for grazing and household sheep (χ^2^ **=** 0.65, *p* > 0.05) (Table 2).

**Table 2:** Prevalence and genotype distribution of *E. bieneusi* by age, gender, and feeding habits in goats and sheep

Age group			<3 m	3–6 m	6 m–1y	>1y	Subtotal	Genotypes (*n* ^a^)
			(b/c) d)	(b/c d)	(b/c d)	(b/c d)	(b/cd)	
Goats	Gender	♀	6/36	16/35	14/49	59/212	95/332	BEB6(23), D(2), E(6), F(2), J(1), KIN-1(1), CD6(7), COS-I(1), CHG1(13),CHG2(2), CHG3(10) ,CHG5(6), CHG6(1), CHG9(1), CHG11(1), CHG13(1), CHG14(1), CHG16(2), CHG18(1),CHG19(1), CHG23(1), CHG24(1), CHG25(1);
			(16.7)	(45.7)	(28.6)	(27.8)	(28.6)	
		♂	12/46	7/26	3/16	59/191	81/279	BEB6(18), D(1), E(2), F(2), KIN-1(1), CD6(2), COS-I(1), CHG1(6), CHG2(4), CHG3(7), CHG5(1), CHG7(1), CHG8(1), CHG10(1), CHG12(1), CHG17(1), CHG20(1), CHG21(1),CHG22(1);
			(26.1)	(26.9)	(18.8)	(30.9)	(29.0)	
	Feeding habits	Grazing	5/24	0/0	7/34	61/242	73/300	BEB6(21), D(3), E(7), F(2), J(1), COS-I(1), CD6(5), CHG1(5), CHG2(1), CHG3(12), CHG5(3), CHG16(1), CHG17(1), CHG19(1), CHG21(1), CHG22(1), CHG25(1);
			(20.8)	(0)	(20.6)	(25.2)	(24.3)	
		Household	13/58	23/61	10/31	57/161	103/311	BEB6(20), E(1), F(1), KIN-1(2), COS-I(1), CD6(4), CHG1(14), CHG2(5), CHG3(5), CHG5(4),CHG6(1), CHG7(1), CHG8(1), CHG9(1), CHG10(1), CHG11(1), CHG12(1), CHG13(1) ,CHG14(1), CHG16(1), CHG18(1), CHG20(1), CHG23(1) ,CHG24(1);
			(22.4)	(37.7)	(32.3)	(35.4)	(33.1)	
sheep	Gender	♀	0/0	26/45	2/9	74/172	106/226	BEB6(27), CHG3(4), COS-I(7), CHS3(2), CHS4(1), CHS7(1), CHS8(1), CHS9(1), CHS11(1);
			(0)	(57.8)	(22.2)	(43.0)	(46.9)	
		♂	13/25	19/41	18/43	25/79	75/188	BEB6(33), CM4(1), COS-I(7), CHG3(1), CHS5(1), CHS6(1), CHS10(1), CHS12(1);
			(52.0)	(46.3)	(45.9)	(31.7)	(39.9)	
	Feeding habits	Grazing	0/0	15/21	11/22	84/205	110/248	BEB6(17), COS-I(5), CM4(1), CHG3(5), CHS4(1);
			(0)	(71.4)	(50.0)	(41.0)	(44.4)	
		Household	13/25	30/65	9/30	15/46	67/166	BEB6(43), COS-I(9), CHS3(2), CHS5(1), CHS6(1), CHS7(1), CHS8(1), CHS9(1), CHS10(1) ,CHS11(1), CHS12(1);
			(52.0)	(46.2)	(30.0)	(32.6)	(40.4)	

*n* ^a^, number of genotypes; ♀: female sign; ♂: male

b/c, No.positive/ No.examined; d, %

### Distribution of *E. bieneusi* genotypes in goats and sheep

A total of 42 ITS genotypes were obtained from 230 successfully sequenced specimens from goats and sheep. Of them, nine genotypes have been previously reported, including BEB6 (EU153584), D (AF101200, syn. CEbC; PigEBITS9; VI; WL18; Peru 9; PtEb), EbpC (AF135832, syn. E; WL13; Peru4; WL17), F (AF135833, syn. EbpA), KIN-1(JQ437573), J(AF135837), COS-I(KJ850432, syn. CM7), CM4(KF543866) and CD6(KJ668733), while other 33 genotypes were novel (CHG1–CHG3, CHG5–CHG14, CHG16–CHG25 and CHS3–CHS12). A higher sequence polymorphism was identified in goat isolates compared to sheep specimens. Of the 139 goat isolates that were successfully sequenced, 31 genotypes were identified, eight of which were known (BEB6, D, EbpC, F, KIN-1, J, COS-I and CD6), while other 23 were new genotypes (CHG1–CHG3, CHG5–CHG14, CHG16–CHG25). For sheep, 91 specimens were sequenced successfully and 14 genotypes were identified, including three known genotypes (BEB6, CM4 and COS-I) and 11 novel genotypes (CHG3 and CHS3–CHS12). Genotypes BEB6, COS-I and CHG3 were found in both sheep and goat specimens (Tables 1, 2, 3).

**Table 3:** Prevalence and ITS genotype distribution of *E. bieneusi* isolates by age group in goats and sheep

Species	Age group	(No. positive/No.examined) (%)	Genotypes (*n* ^a^)
Goats	<3 m	18/82(22.0)	CHG1(1), CHG2(1), CD6(4), CHG5(1);
	3-6 m	23/61(37.7)	BEB6(4), F(1), CD6(2), CHG1(5), CHG2(2), CHG3(3), CHG5(2), CHG14(1);
	6 m-1y	17/65(26.2)	BEB6(10), E(2), CHG13(1), CHG18(1);
	>1y	118/403(29.3)	BEB6(27), D(3), E(6), F(3), J(1), KIN-1(2), CD6(3), COS-I(2), CHG1(13), CHG2(3), CHG3(14), CHG5(4), CHG6(1), CHG7(1), CHG8(1), CHG9(1), CHG10(1), CHG11(1), CHG12(1), CHG16(2), CHG17(1), CHG19(1), CHG20(1), CHG21(1), CHG22(1), CHG23(1), CHG24(1), CHG25(1);
	Subtotal	176/611(28.8)	BEB6(41), D(3), E(8), F(4), KIN-1(2), J(1), CD6(9), COS-I(2), CHG1(19), CHG2(6), CHG3(17), CHG5(7), CHG6(1), CHG7(1), CHG8(1), CHG9(1), CHG10(1), CHG11(1), CHG12(1), CHG13(1), CHG14(1), CHG16(2), CHG17(1), CHG18(1), CHG19(1), CHG20(1), CHG21(1), CHG22(1), CHG23(1), CHG24(1), CHG25(1);
Sheep	<3 m	13/25(52.0)	BFB6(9), CHS6(1), COS-I(2);
	3-6 m	45/86(52.3)	BFB6(26), COS-I(6), CHG3(2), CHS3(1), CHS4(1), CHS5(1), CHS7(1), CHS8(1), CHS9(1), CHS11(1);
	6 m-1y	20/52(38.5)	BEB6(7), CHS10(1), CHS12(1);
	>1y	99/251(39.4)	BEB6(18), CM4(1), COS-I(6) ,CHG3(3), CHS3(1);
	Subtotal	177/414(42.8)	BEB6(60), CHG3(5), COS-I(14), CM4(1), CHS3(2), CHS4(1), CHS5(1), CHS6(1), CHS7(1), CHS8(1), CHS9(1), CHS10(1), CHS11(1), CHS12(1);
Total		353/1025(34.4)	F(4), KIN-1(2), J(1), CHG1(19), CHG2(6), CHG3(22), CD6(9), CHG5(7), CHG6(1), CHG7(1), CHG8(1), CHG9(1), CHG10(1),CHG11(1), CHG12(1), CHG13(1), CHG14(1), COS-I(16), CHG16(2), CHG17(1), CHG18(1), CHG19(1), CHG20(1), CHG21(1), CHG22(1), CHG24(1),CHG25(1), CM4(1), CHS3(2), CHS4(1), CHS5(1), CHS6(1), CHS7(1), CHS8(1), CHS9(1), CHS10(1), CHS11(1), CHS12(1), BEB6(101), D(3), E(8), CHG23(1)

*n* ^a^, number of genotypes

The genotype BEB6 was the dominant genotype in goat (41/139) and sheep isolates (60/91) in this study. In goats, in addition to BEB6, the other dominant genotypes, namely CHG1 and CHG3, were detected in 19 and 17 specimens, respectively. The genotypes CD6, CHG5, EbpC, CHG2, F, and D were identified in nine, seven, eight, six, four, and three specimens, respectively. Genotypes KIN-1, COS-I and CHG16 were observed in two specimens each, while all other genotypes we identified were found in one specimen each. In sheep, the genotype COS-I was the second dominant genotype, being identified in 14 specimens. Genotypes CHG3 and CHS2 were identified in five and two specimens, respectively, while all other genotypes we obtained were identified in one specimen each. The genotype distribution according to the age, gender and the feeding habits of the animals is shown in Tables 2 and 3.

### Phylogenetic analysis

Phylogenetic analysis revealed that some novel genotypes from this study belonged to the human-pathogenic group [15], recently renamed group 1 [39, 40]. We identified genotypes D, KIN-1, EbpC and F reported previously and 10 novel genotypes (CHG6, CHG7, CHG9, CHG19, CHG23, CHG25, CHS5, CHS10, CHS11 and CHS12) belonged to this group. The novel genotypes CHG23, CHG25, CHS10 and CHG19 had one (C to A substitution at nucleotide position 362), two (T to C and T to A substitutions at positions 243 and 245), two (T to G substitutions at positions 223 and 252) and three (A to G, T to G and G to A substitutions at positions 137, 201 and 374) single nucleotide polymorphisms (SNPs) compared with the established genotype, EbpC (AF135832). Therefore, genotypes CHG23, CHG25, NESH1, CHS10 and CHG19 were clustered into subgroup 1d with the EbpC genotype. The following new genotypes CHS5, CHS11 and CHS12 were grouped with subgroup 1e with known genotypes COG-1 (KJ850434) and F (AF135833). Compared with the known genotype F (AF135833), one or several SNPs were present (data not shown). The new genotype CHG9 differed from the genotype C (AF101199) at nucleotide positions 123 (G to A substitution), 230 (G to A substitution), 255(G to T substitution) and 284 (G to A substitution), respectively, thus they formed subgroup 1 f. However, the new genotypes CHG6 and CHG7 formed a cluster distinct from any known subgroup 1. We found that 22 new genotypes (CHG1–CHG3, CHG5, CHG8, CHG10–CHG14, CHG16–CHG18, CHG20, CHG22, CHG24, CHS3, CHS4, and CHS6–CHS9) and 7 known genotypes (CD6, COS-I, BEB6, J, COS-III, NESH4 and CHN2) belonged to the host-specific group 2, but the known genotypes J (AF135837) and CHN2 (HM992510) formed one branch, and the 22 new genotypes with the reported genotypes CD6(KJ668733), COS-I(KJ850432),COS-III(KP063053), NESH4(KP732478) and BEB6(EU153584) formed another clade. Compared with genotype BEB6 (EU153584), new genotypes CHG2, CHG13, CHG16, CHG17, CHS3, CHS4, CHS7, CHS8 and CHS9 contained one SNP, genotypes CHG3, CHG5, CHG12, CHG22 and CHS6 contained two SNPs, genotypes CHG1, CHG14, CHG18 and CHG20 contained three SNPs, and genotypes CHG11, CHG10 and CHG24 contained 4, 11 and 13 SNPs, respectively, the genotype CHG8 contained over 15 SNPs (Additional file 2). However, the new genotype CHG21 was clustered with the CM4 (KF543866) genotype [20, this study] in the group 9 [41] ( Additional file 3: Figure S1).

## Discussion

In the present study, *E. bieneusi* was found to be a common parasite in sheep and goats. The prevalence of this organism in healthy sheep and goats was 42.8 % and 28.8 %, respectively. A similar prevalence (21.8 %) was reported for *E. bieneusi* in goats in Heilongjiang Province [37], while lower prevalence rates (22.5 % and 13.9 %) were detected in Heilongjiang Province [37] and northeast China [35] in sheep. A higher *E. bieneusi* prevalence (69.3 %) was found in sheep in Inner Mongolia [34]. The differences in the prevalence between this study and others [6, 9, 34, 35, 37] are likely due to differences in the age groups studied, as well as the number of study animals, detection methods, and the geographic and ecological environments. Here we described the infection rates of *E. bieneusi* in different age groups in sheep and goats and showed that there were no significant differences among different age groups, which was consistent with the conclusion obtained by Jiang and others [35] but inconsistent with the results gained by Stensvold [10]. In this study, we have described the prevalence of *E. bieneusi* by the gender and different feeding habits of the host animals for the first time in detail, although there were no significant differences between them. Nevertheless, these results provide an useful reference data for researchers in this field.

All nine known genotypes identified in the present study have been previously reported in China: the genotype BEB6 has been reported in one child (reported as SH5, [30]), sheep [9], NHPs [20, 42], deer [36], cats [12] and golden takins [43], even in urban wastewater [38]. This genotype was first detected in cattle in the United States [13] and is considered to be a cattle-adopted genotype. However, the genotype BEB6 was detected in 43.9 % (101/230) sequenced specimens and found in almost every farm (Additional file 1), which showed that this genotype was observed as a dominant genotype with a wide geographic distribution, extensive host range and zoonotic potential both in the present study and in other reports [9, 10, 23, 34–37]. The genotype D has been found in AIDS patients [30], children [31], NHPs [33, 42], pigs [8], dogs [12, 44], cats [12, 44], foxes [45], raccoon dogs [45], cattle [46], golden takins [43], urban wastewater [38] and drinking source watershed [47]. In this report, this genotype was only found in goats, in contrast to the results reported in Heilongjiang Province [37]. Though the genotype D only infected goats in this study, the transmission source and transmission dynamics of this genotype is still unclear and further research is required to understand the role of this genotype in microsporidiosis. The genotype EbpC has been found in HIV-positive and -negative patients [30], children [31], NHPs [20, 33], dogs [12, 44], pigs [8, 9], lake water [32], red pandas [48] and urban wastewater [38]. Here, the genotype EbpC was detected only in goat specimens, which was similar to the results of Zhao et al [37], but was in contrast with Li et al [35]. The genotype F has been found in children [31], pigs [8], cattle [46] and wastewater [38]. Genotypes J has been reported in humans [11], cattle [49] and yaks [50], KIN-1 in humans (JQ437573, unpublished), CD6(KJ668733), COS-I(KJ850432) (syn. CM7) in sheep [35, 37], deer [51] and NHPs [20], CM4 in NHPs [20] and CD6 in dogs [20].

Among the nine established genotypes found in this study, six (BEB6, D, EbpC, F, KIN-1, J) genotypes have been reported in humans [11, 30, 31]. Thus, some of the genotypes in sheep and goats have zoonotic potential, and sheep and goats may be reservoirs of human microsporidiosis.

Phylogenetic analysis showed that 10 new *E. bieneusi* genotypes (CHG6, CHG7, CHG9, CHG19, CHG23, CHG25, CHS5, CHS10, CHS11, and CHS12) belonged to the so-called group 1 with zoonotic potential [39]. This finding merits close attention because of the ability of these genotypes to cause *E. bieneusi*-related microsporidiosis in humans. The other 22 new genotypes (CHG1–CHG3, CHG5, CHG8, CHG10–CHG14, CHG16–CHG18, CHG20, CHG22, CHG24, CHS3, CHS4, and CHS6–CHS9) were classified into the cattle-specific group 2 [39]. Some genotypes from this group initially considered to be cattle-specific (such as BEB6 and J), were later found in other animals including humans [11, 12, 20, 30, 50, this research], therefore some genotypes in the group 2 may have limited zoonotic potential and should be examined in more detail. The remaining new genotype CHG21 with only one SNP compared with the genotype CM4 formed a cluster, in the previously described group 9 [41].

## Conclusions

This study to infer the prevalence and genotype distribution of *E. bieneusi* in sheep and goats in China. The data showed there were a widespread distribution, high prevalence, and considerable genetic diversity in *E. bieneusi* genotypes from farmed goats and sheep, which may cause microsporidiosis among sheep, goats, other animals and humans resulting in economic losses in animal husbandry. Moreover, many genotypes from this study have been reported in humans and urban wastewater in China, which may result in zoonotic transmission. Therefore, contact between susceptible human populations and goats or sheep should be avoided to reduce zoonotic or anthroponotic parasite transmission. Further studies are needed to fully elucidate the importance of goats and sheep in the epidemiology of human microsporidiosis.

## Additional files

Additional file 1:
**Prevalence and genotype distribution of** *E. bieneusi* **in goats and sheep on each farm.** (DOC 117 kb) Additional file 2:
**Nucleotide substitutions among some new genotypes of** *E. bieneusi* **from this study versus the reported genotype BEB6 (EU 153584).** (DOC 132 kb) Additional file 3: Figure S1. FIG 1 Phylogenetic relationships of the *E. bieneusi* genotypes identified in this study and other reported genotypes, as inferred by neighbor-joining analysis of ITS gene sequences based on the distances calculated using the Kimura 2-parameter model. Bootstrap values >50 % from 1000 replicates are shown on the nodes. The tree was rooted with GenBank sequence AF059610 from a dog. Known genotypes observed in goats and sheep are marked with open squares and triangles, and the new genotypes in goats and sheep from this study are indicated by filled squares and triangles. (TIF 2078 kb)
