# Supplementary material for: Molecular survey of Enterocytozoon bieneusi in sheep and goats in China
Source: Parasit Vectors. 2016 Jan 19;9:23. doi: 10.1186/s13071-016-1304-0 (PMC5024852; doi:10.1186/s13071-016-1304-0)
Supplement: Additional file 1: — Prevalence and genotype distribution of E. bieneusi in goats and sheep on each farm. (DOC 117 kb) [file 13071_2016_1304_MOESM1_ESM.pdf]

Additional file 1. Prevalence and genotype distribution of *E. bieneusi* isolates from goat and sheep farms in China

Species	Geographica source	Farm	No. of specimens	No. (%) of Positive specimens	ITS genotype(s) (n^a)
**Goats**	Henan Province	Zhengzhou	105	48（45.7）	BEB6(4), F(1), KIN-1(2), CHG1(6), CHG2(4), CHG5(1), CHG6(1), CHG7(1), CHG8(1), CHG9(1),CHG10(1), CHG11(1) CHG20(1), CHG23(1)
		Xinyang	15	8（53.3）	BEB6(1), E(1), CHG3(5)
		Pingdingshan	63	10（15.9）	BEB6(2), E(1), F(1), CHG1(1), CHG5(2), CHG22(1)
		Sanmenxia	24	9（37.5）	BEB6(2), D(2), CHG1(1), CHG3(2), CHG21(1)
		Gongyi	18	7（38.9）	BEB6(1), E(1), J(1), CHG3(2),CHG25(1)
		Nanyang	37	7（18.9）	BEB6(3), CHG1(1), CHG2(1)
		Kaifeng	40	8（20.0）	CHG1(2), CHG2(1), CHG3(1), CD6(4)
		Shangqiu	13	7（53.9）	BEB6(3), CHG1(2), COS-I (1)
		Zhumadian	22	8（36.3）	BEB6(5), CHG13(1), CHG18(1)
		Jiyuan	6	1（16.7）	BEB6(1)
		Subtotal	343	113（32.9）	BEB6(22), E(3), F(2), KIN-1(2), D(2), J(1), COS-I(1), CD6(4), CHG1(13), CHG2(6), CHG3(10), CHG5(3), CHG6(1),CHG7(1),CHG8(1), CHG9(1), CHG10(1), CHG11(1), CHG13(1), CHG18(1), CHG20(1), CHG21(1), CHG22(1), CHG23(1)), CHG25(1),
	Yunnan Province	Wumushan	36	6（16.7）	BEB6(2), D(1), CHG3(1), COS-I(1), CHG16(1)
		Mengbang	30	3（10.0）	BEB6(1), E(1), CHG19(1)
		Hexin	28	7（25.0）	BEB6(5), E(1), CHG17(1)
		Kunming	40	14（35.0）	BEB6(6), E(2), F(1), CHG1(1), CHG3(1), CD6(1),CHG5(1)
		Subtotal	134	30（22.4）	BEB6(14), E(4), F(1), D(1), COS-I(1), CD6(1), CHG1(1), CHG3(2), CHG5(1), CHG16(1), CHG17(1), CHG19(1)
	Anhui Province	Anhui	80	6（7.5）	BEB6(1), CHG5(1), CHG3(1)
	Chongqi city	Chongqi	8	5（62.5）	CHG1(2), CHG3(1), CD6(1), CHG12(1)

	Shaanxi Province	Xi’an	46	22（47.8）	BEB6(4), E(1), F(1), CHG1(3), CHG3(3), CD6(3), CHG5(2), CHG14(1), CHG16(1), CHG24(1)
	Total		611	176（28.8）	BEB6(41), D(3), E(8), F(4), KIN-1(2), J(1), CHG1(19), CHG2(6), CHG3(17), CD6(9), CHG5(7), CHG6(1), CHG7(1), CHG8(1), CHG9(1), CHG10(1), CHG11(1), CHG12(1), CHG13(1), CHG14(1), COS-I(2), CHG16(2), CHG17(1), CHG18(1),CHG19(1), CHG20(1), CHG21(1), CHG22(1), CHG23(1), CHG24(1), CHG25(1)
**Sheep**	Henan Province	Anyang	30	13（43.3）	BEB6(9), COS-I(2), CHS6(1)
		Dengfeng	49	33（67.4）	BEB6(21), COS-I (5), CHS3(2), CHS5(1)
		Pidingshan	47	11(23.4)	BEB6(9), CHS10(1), CHS12(1)
		Zhengzhou	184	104(56.5)	BEB6(14), CM4(1), COS-I (5), CHG3(5), CHS4(1)
		Subtotal	310	161（51.9）	BEB6(53), COS-I (12), CM4(1), CHG3(5), CHS3(2), CHS4(1), CHS5(1), CHS6(1), CHS10(1), CHS12(1)
	Liaoning Province	Liaoning	64	6 (9.4)	BEB6(3)
	Heilongjian Province	Qiqihar	40	10 (25.0)	BEB6(4), COS-I (2), CHS7(1), CHS8(1), CHS9(1), CHS11(1)
	Total		414	177 (42.8)	BEB6(60), COS-I (14), CHG3(5), CM4(1), CHS3(2), CHS4(1), CHS5(1), CHS6(1), CHS7(1), CHS8(1),CHS9(1),CHS10(1), CHS11(1), CHS12(1)
	All the Total		1025	353(34.4)	F(4), KIN-1(2), J(1), CHG1(19), CHG2(6), CHG3(22), CD6(9), CHG5(7), CHG6(1), CHG7(1), CHG8(1),CHG9(1), CHG10(1), CHG11(1), CHG12(1), CHG13(1), CHG14(1), COS-I(16), CHG16(2), CHG17(1),CHG18(1), CHG19(1),CHG20(1), CHG21(1), CHG22(1), CHG23(1), CHG24(1), CHG25(1), CM4(1), CHS3(2), CHS4(1), CHS5(1), CHS6(1),CHS7(1), CHS8(1), CHS9(1), CHS10(1), CHS11(1), CHS12(1), BEB6(101), D(3), E(8)

n[a], number of genotypes.
