# Supplementary material for: Molecular survey of Enterocytozoon bieneusi in sheep and goats in China
Source: Parasit Vectors. 2016 Jan 19;9:23. doi: 10.1186/s13071-016-1304-0 (PMC5024852; doi:10.1186/s13071-016-1304-0)
Supplement: Additional file 2: — Nucleotide substitutions among some new genotypes of E. bieneusi from this study versus the reported genotype BEB6 (EU 153584). (DOC 132 kb) [file 13071_2016_1304_MOESM2_ESM.pdf]

Additional file 2. Nucleotide substitutions among novel genotypes from this study versus the reported reference genotype BEB6 (EU153584), which belongs to group 2

Genotypes	Nucleotide positions and substitutions																													
	35	44	77	86	90	94	108	109	121	127	134	150	152	158	171	181	193	205	207	213	225	226	249	254	255	256	261	263	265	267	270
BEB6	C	G	T	A	T	A	T	A	A	G	T	C	C	G	G	T	T	A	C	T	G	G	G	A	C	G	G	T	G	T	A
CHG1	-	-	-	-	-	-	C	-	-	-	G	-	-	-	-	-	-	-	-	-	-	-	-	-	-	-	-	-	T	-	-
CHG2	-	-	-	-	-	-	-	-	-	-	G	-	-	-	-	-	-	-	-	-	-	-	-	-	-	-	-	-	-	-	-
CHG3	-	-	-	-	-	-	-	-	-	-	G	-	-	-	-	C	-	-	-	-	-	-	-	-	-	-	-	-	-	-	-
CHG5	-	-	-	-	-	-	-	-	-	-	G	-	-	-	-	-	-	-	-	-	-	-	-	-	-	-	-	-	T	-	-
CHG8	-	-	-	G	-	G	-	G	G	-	G	-	-	-	T	-	G	G	G	C	-	-	-	G	T	-	T	G	A	G	-
CHG10	-	-	-	G	-	G	-	G	-	-	G	-	-	-	-	-	G	G	G	C	-	-	-	-	-	-	-	G	A	G	-
CHG11	-	-	-	G	-	-	-	G	-	-	-	-	-	-	-	-	-	G	G	-	-	-	-	-	-	-	-	-	-	-	-
CHG12	T	-	-	-	-	-	-	-	-	-	G	-	-	-	-	-	-	-	-	-	-	-	-	-	-	-	-	-	-	-	-
CHG13	-	-	-	-	-	-	-	G	-	-	-	-	-	-	-	-	-	-	-	-	-	-	-	-	-	-	-	-	-	-	-
CHG14	T	-	-	-	-	-	C	-	-	-	G	-	-	-	-	-	-	-	-	-	-	-	-	-	-	-	-	-	-	-	-
CHG16	-	-	-	-	-	-	-	-	-	-	-	-	-	A	-	-	-	-	-	-	-	-	-	-	-	-	-	-	-	-	-
CHG17	-	-	-	-	-	-	-	-	-	-	-	-	-	-	-	-	-	-	-	-	-	A	-	-	-	-	-	-	-	-	-
CHG18	-	-	-	-	-	-	-	-	-	-	-	-	-	-	T	-	-	G	-	-	-	-	-	-	-	-	-	-	-	G	-
CHG20	-	-	-	-	-	-	-	-	-	-	G	-	-	-	-	-	-	-	-	-	-	-	-	G	T	-	-	-	-	-	-
CHG22	-	-	C	-	-	-	-	-	-	-	-	-	T	-	-	-	-	-	-	-	-	-	-	-	-	-	-	-	-	-	-
CHG24	-	-	-	G	-	-	-	-	-	-	G	A	-	-	-	-	-	G	G	C	-	-	-	G	T	-	T	G	A	G	G
CHS3	-	-	-	-	-	-	-	-	-	-	-	-	-	-	-	-	-	-	-	-	-	-	A	-	-	-	-	-	-	-	-
CHS4	-	T	-	-	-	-	-	-	-	-	-	-	-	-	-	-	-	-	-	-	-	-	-	-	-	-	-	-	-	-	-
CHS6	-	-	-	-	C	-	-	-	-	-	-	-	-	-	-	-	-	-	-	-	-	-	A	-	-	-	-	-	-	-	-

CHS7	-	-	-	-	-	-	-	-	-	A	-	-	-	-	-	-	-	-	-	-	-	-	-	-	-	-	-	-	-	-	-
CHS8	-	-	-	-	-	-	-	-	-	-	-	-	-	-	-	-	-	-	-	-	A	-	-	-	-	-	-	-	-	-	-
CHS9	-	-	-	-	-	-	-	-	-	-	-	-	-	-	-	-	-	-	-	-	-	-	-	-	-	A	-	-	-	-	-

-, the same base pair.
